# Supplementary material for: Mitochondrial integrated stress response controls lung epithelial cell fate
Source: Nature. 2023 Aug 9;620(7975):890–7. doi: 10.1038/s41586-023-06423-8 (PMC10447247; doi:10.1038/s41586-023-06423-8)
Supplement: Supplementary file 1 — . [file 41586_2023_6423_MOESM1_ESM.pdf]

nature portfolio

https://doi.org/10.1038/s41586-023-06423-8

Supplementary information

Mitochondrial integrated stress response controls lung epithelial cell fate

In the format provided by the authors and unedited

Supplementary Table 1. Mouse integrated stress response (ISR) associated genes.

Group	Gene ID	Symbol
gene.clic	ENSMUSG00000032715	*Trib3*
gene.clic	ENSMUSG00000027313	*Chac1*
gene.clic	ENSMUSG00000005667	*Mthfd2*
gene.clic	ENSMUSG00000040010	*Slc7a5*
gene.clic	ENSMUSG00000031490	*Eif4ebp1*
gene.clic	ENSMUSG00000025408	*Ddit3*
gene.clic	ENSMUSG00000020142	*Slc1a4*
gene.clic	ENSMUSG00000038539	*Atf5*
gene.clic	ENSMUSG00000028893	*Sesn2*
gene.clic	ENSMUSG00000029752	*Asns*
gene.clic	ENSMUSG00000029777	*Gars*
gene.clic	ENSMUSG00000031770	*Herpud1*
gene.clic	ENSMUSG00000040435	*Ppp1r15a*
gene.clic	ENSMUSG00000026628	*Atf3*
gene.clic	ENSMUSG00000036390	*Gadd45a*
gene.clic	ENSMUSG00000001918	*Slc1a5*
gene.clic	ENSMUSG00000026663	*Atf6*
gene.clic	ENSMUSG00000010095	*Slc3a2*
gene.clic	ENSMUSG00000026456	*Cyb5r1*
gene.clic	ENSMUSG00000025007	*Aldh18a1*
gene.clic	ENSMUSG00000026142	*Rhbdd1*
gene.clic	ENSMUSG00000030717	*Nupr1*
gene.clic	ENSMUSG00000053398	*Phgdh*
gene.clic	ENSMUSG00000027737	*Slc7a11*
gene.clic	ENSMUSG00000005413	*Hmox1*
gene.clic	ENSMUSG00000023067	*Cdkn1a*
gene.clic	ENSMUSG00000024587	*Nars*
gene.clic	ENSMUSG00000031960	*Aars*
gene.clic	ENSMUSG00000029446	*Psph*
gene.clic	ENSMUSG00000068739	*Sars*
gene.clic	ENSMUSG00000037851	*Iars*
gene.clic	ENSMUSG00000024493	*Lars*
gene.clic	ENSMUSG00000028542	*Slc6a9*
gene.clic	ENSMUSG00000028811	*Yars*
gene.clic	ENSMUSG00000028179	*Cth*
gene.clic	ENSMUSG00000020256	*Aldh1l2*
gene.clic	ENSMUSG00000040618	*Pck2*
gene.clic	ENSMUSG00000031700	*Gpt2*

gene.clic	ENSMUSG00000023883	*Phf10*
gene.clic	ENSMUSG00000022241	*Tars*
gene.clic	ENSMUSG00000030738	*Eif3c*
gene.clic	ENSMUSG00000027944	*Hax1*
gene.clic	ENSMUSG00000025403	*Shmt2*
gene.clic	ENSMUSG00000025140	*Pycr1*
gene.clic	ENSMUSG00000021831	*Ero1l*
gene.clic	ENSMUSG00000041168	*Lonp1*
gene.clic	ENSMUSG00000074656	*Eif2s2*
gene.clic	ENSMUSG00000040354	*Mars*
gene.clic	ENSMUSG00000034667	*Xpot*
gene.clic	ENSMUSG00000038742	*Angptl6*
gene.clic	ENSMUSG00000031513	*Leprotl1*
gene.clic	ENSMUSG00000041028	*Ghitm*
gene.clic	ENSMUSG00000024359	*Hspa9*
gene.clic	ENSMUSG00000038615	*Nfe2l1*
gene.clic	ENSMUSG00000021203	*Otub2*
gene.clic	ENSMUSG00000025190	*Got1*
gene.clic	ENSMUSG00000038508	*Gdf15*
gene.clic	ENSMUSG00000028982	*Slc25a33*
gene.clic	ENSMUSG00000001627	*Ifrd1*
gene.clic	ENSMUSG00000023952	*Gtpbp2*
gene.clic	ENSMUSG00000023045	*Soat2*
gene.clic	ENSMUSG00000056749	*Nfil3*
gene.clic	ENSMUSG00000036432	*Siah2*
gene.clic	ENSMUSG00000027357	*Crls1*
gene.clic	ENSMUSG00000042747	*Krtcap2*
gene.clic	ENSMUSG00000039512	*Uhrf1bp1*
gene.clic	ENSMUSG00000032252	*Glce*
gene.clic	ENSMUSG00000028059	*Arhgef2*
gene.clic	ENSMUSG00000056501	*Cebpb*
gene.clic	ENSMUSG00000026483	*Fam129a*
gene.clic	ENSMUSG00000023977	*Ubr2*
gene.clic	ENSMUSG00000036693	*Nop14*
gene.clic	ENSMUSG00000022365	*Derl1*
gene.clic	ENSMUSG00000031297	*Slc7a3*
gene.clic	ENSMUSG00000038550	*Ciart*
gene.clic	ENSMUSG00000040511	*Pvr*
gene.clic	ENSMUSG00000023951	*Vegfa*
gene.clic	ENSMUSG00000040675	*Mthfd1l*
gene.clic	ENSMUSG00000037242	*Clic4*

gene.clic	ENSMUSG00000000278	*Scpep1*
gene.clic	ENSMUSG00000018848	*Rars*
gene.clic	ENSMUSG00000022369	*Mtbp*
gene.clic	ENSMUSG00000021196	*Pfkp*
gene.clic	ENSMUSG00000033813	*Tcea1*
gene.clic	ENSMUSG00000037722	*Gnpnat1*
gene.clic	ENSMUSG00000028972	*Car6*
gene.clic	ENSMUSG00000026317	*Cln8*
gene.clic	ENSMUSG00000041390	*Mdfic*
gene.clic	ENSMUSG00000022364	*Tbc1d31*
gene.clic	ENSMUSG00000047407	*Tgif1*
gene.clic	ENSMUSG00000055725	*Paqr3*
gene.clic	ENSMUSG00000020108	*Ddit4*
gene.clic	ENSMUSG00000015652	*Steap1*
gene.clic	ENSMUSG00000054136	*Adm2*
gene.clic	ENSMUSG00000024664	*Fads3*
extended	ENSMUSG00000042406	*Atf4*
extended	ENSMUSG00000042750	*Bex2*
extended	ENSMUSG00000036478	*Btg1*
extended	ENSMUSG00000010755	*Cars*
extended	ENSMUSG00000024039	*Cbs*
extended	ENSMUSG00000071470	*Ccnb1ip1*
extended	ENSMUSG00000034563	*Ccpg1*
extended	ENSMUSG00000056216	*Cebpg*
extended	ENSMUSG00000048065	*Cyb5r2*
extended	ENSMUSG00000035530	*Eif1*
extended	ENSMUSG00000006941	*Eif1b*
extended	ENSMUSG00000031490	*Epb41l4a*
extended	ENSMUSG00000043068	*Fam89a*
extended	ENSMUSG00000030827	*Fgf21*
extended	ENSMUSG00000020875	*Hoxb9*
extended	ENSMUSG00000025950	*Idh1*
extended	ENSMUSG00000028266	*Lmo4*
extended	ENSMUSG00000052727	*Map1b*
extended	ENSMUSG00000024235	*Map3k8*
extended	ENSMUSG00000020000	*Moxd1*
extended	ENSMUSG00000039670	*Oxld1*
extended	ENSMUSG00000024014	*Pim1*
extended	ENSMUSG00000070366	*Plpp4*
extended	ENSMUSG00000024640	*Psat1*

extended	ENSMUSG00000026475	*Rgs16*
extended	ENSMUSG00000041771	*Slc24a4*
extended	ENSMUSG00000041313	*Slc7a1*
extended	ENSMUSG00000104960	*Snhg8*
extended	ENSMUSG00000020027	*Socs2*
extended	ENSMUSG00000009588	*St6galnac1*
extended	ENSMUSG00000031431	*Tsc22d3*
extended	ENSMUSG00000021266	*Wars*
extended	ENSMUSG00000041774	*Ydjc*
extended	ENSMUSG00000039501	*Znfx1*
