# Supplementary material for: Mitochondrial integrated stress response controls lung epithelial cell fate
Source: Nature. 2023 Aug 9;620(7975):890–7. doi: 10.1038/s41586-023-06423-8 (PMC10447247; doi:10.1038/s41586-023-06423-8)
Supplement: Supplementary file 2 — Reporting Summary [file 41586_2023_6423_MOESM2_ESM.pdf]

nature portfolio

Corresponding author(s): SeungHye Han, Navdeep Chandel

Last updated by author(s): Jun 5, 2023

Reporting Summary

Nature Portfolio wishes to improve the reproducibility of the work that we publish. This form provides structure for consistency and transparency in reporting. For further information on Nature Portfolio policies, see our Editorial Policies and the Editorial Policy Checklist.

Statistics

For all statistical analyses, confirm that the following items are present in the figure legend, table legend, main text, or Methods section.

n/a	Confirmed	
☐	☒	The exact sample size (*n*) for each experimental group/condition, given as a discrete number and unit of measurement
☐	☒	A statement on whether measurements were taken from distinct samples or whether the same sample was measured repeatedly
☐	☒	The statistical test(s) used AND whether they are one- or two-sided<br>*Only common tests should be described solely by name; describe more complex techniques in the Methods section.*
☐	☒	A description of all covariates tested
☐	☒	A description of any assumptions or corrections, such as tests of normality and adjustment for multiple comparisons
☐	☒	A full description of the statistical parameters including central tendency (e.g. means) or other basic estimates (e.g. regression coefficient) AND variation (e.g. standard deviation) or associated estimates of uncertainty (e.g. confidence intervals)
☐	☒	For null hypothesis testing, the test statistic (e.g. *F*, *t*, *r*) with confidence intervals, effect sizes, degrees of freedom and *P* value noted<br>*Give P values as exact values whenever suitable.*
☒	☐	For Bayesian analysis, information on the choice of priors and Markov chain Monte Carlo settings
☐	☒	For hierarchical and complex designs, identification of the appropriate level for tests and full reporting of outcomes
☐	☒	Estimates of effect sizes (e.g. Cohen's *d*, Pearson's *r*), indicating how they were calculated

Our web collection on statistics for biologists contains articles on many of the points above.

Software and code

Policy information about availability of computer code

Data collection	Oxygen consumption data was collected using Wave 2.6.3.5 software. Immunoblot data was collected using a Wes by ProteinSimple and Compass for SW software 5.0.1. Nikon A1C confocal microscope, Nikon Ti2 Widefield microscope, and TissueGnostics imaging software system (TissueFAXS 7.1) were used to obtain images. RNA-seq data was collected using Illumina NextSeq 500 system. Raw BCL read files were demultiplexed using bcl2fastq V2.20.0 (Illumina), and trimmed using Trimmomatic (version 0.39). For metabolomics, high-resolution HPLC–tandem mass spectrometry was performed on a Q-Exactive (ThermoFisher Scientific) in line with an electrospray source and an UltiMate 3000 (ThermoFisher Scientific) and data were collected using Xcalibur 4.1 software. Single-cell RNA-seq data was obtained from HiSeq 4000 instrument (Illumina), and raw sequencing reads were processed using CellRanger v6.0.1.
Data analysis	Image processing and analysis was performed using freely (ImageJ/Fiji 1.53 [NIH]) or commercially available software (Nikon Elements (5.11.00)). Immunoblot data were analyzed using Compass for SW software 5.0.1 (ProteinSimple).<br>RNA-seq data was analyzed using the R package edgeR. Reads were then aligned to the GRCm39 reference genome using the STAR aligner V2.7.7, and counts were calculated using HTseq V0.11.0. The ComBat-seq package was used to adjust for batch effect on RNA-seq count data. Metabolomic data were analyzed using the MetaboAnalyst software V5.0 and the MetaboAnalystR package V4.1.2. Single-cell RNA-seq data analyses were performed using Seurat v4.0.6 in R v4.1.2 and Scanpy v1.8.1 in Python v3.8.3. Doublets were removed using Scrublet v0.2.1 from each library. RNA velocity was calculated with velocyto v0.17 and scVelo v0.2.4. UCell algorithm was used to evaluate gene signature in single-cell datasets. All code used for analysis is available at https://github.com/MinhoLee-DGU/2023.Han.et.al.Nature<br>All other statistical analyses were performed using GraphPad Prism 9.5.0.

For manuscripts utilizing custom algorithms or software that are central to the research but not yet described in published literature, software must be made available to editors and reviewers. We strongly encourage code deposition in a community repository (e.g. GitHub). See the Nature Portfolio guidelines for submitting code & software for further information.

Data

Policy information about availability of data

All manuscripts must include a data availability statement. This statement should provide the following information, where applicable:

- Accession codes, unique identifiers, or web links for publicly available datasets
- A description of any restrictions on data availability
- For clinical datasets or third party data, please ensure that the statement adheres to our policy

All raw sequencing data (.fastq) generated in this study are available at the NCBI BioProject with the following Accession IDs: PRJNA865889, PRJNA940730, PRJNA940746, PRJNA940973, PRJNA940986, and PRJNA940992.
Strunz et al. (GSE141259), Choi et al. (GSE145031), Kobayashi et al. (GSE141634), Negretti et al. (PRJNA674755 and PRJNA693167), Hurskainen et al. (PRJNA637911), Molecular Signatures Database (MSigDB), and GRCm39 reference genome were used for analysis.

Field-specific reporting

Please select the one below that is the best fit for your research. If you are not sure, read the appropriate sections before making your selection.

☒ Life sciences ☐ Behavioural & social sciences ☐ Ecological, evolutionary & environmental sciences

For a reference copy of the document with all sections, see nature.com/documents/nr-reporting-summary-flat.pdf

Life sciences study design

All studies must disclose on these points even when the disclosure is negative.

Sample size	All experiments were performed using sample sizes based on standard protocols in the field. We made every effort to avoid excessive or needless use of animals. No statistical tests were used to predetermine sample sizes. We used sample sizes commonly used in literature in the field. We used statistical analysis consistent with the sample size for each experiment and found sufficient statistical power with the sample sizes used in our study.
Data exclusions	No animal data were excluded from analyses.<br>Single-cell RNA-seq: Poor quality cells with less than 500 detected genes and a high percentage of mitochondrial genes (>25%) were excluded from analyses.
Replication	All experimental data were reliably reproduced in multiple independent experiments as indicated in the figure legends. For in vivo experiments, multiple mice were used in at least two independent cohorts to ensure reproducibility.
Randomization	Experiments were not randomized. Transgenic mice were predetermined by mouse genotype and therefore could not be randomized. All mice were sex- and age-matched, and littermates when possible.
Blinding	Investigators were not blinded. Blinding was not relevant in this study, as groups consisted of previously genotyped mice or treated cell lines in order to have correct experimental and control groups.

Reporting for specific materials, systems and methods

We require information from authors about some types of materials, experimental systems and methods used in many studies. Here, indicate whether each material, system or method listed is relevant to your study. If you are not sure if a list item applies to your research, read the appropriate section before selecting a response.

Materials & experimental systems

n/a	Involved in the study	
☐	☒	Antibodies
☐	☒	Eukaryotic cell lines
☒	☐	Palaeontology and archaeology
☐	☒	Animals and other organisms
☒	☐	Human research participants
☒	☐	Clinical data
☒	☐	Dual use research of concern

Methods

n/a	Involved in the study	
☒	☐	ChIP-seq
☒	☐	Flow cytometry
☒	☐	MRI-based neuroimaging

Antibodies

Antibodies used	Antibodies used for immunoblot: anti-Vinculin (abcam, ab129002, clone EPR8185; 1:500 dilution); anti-NDUFS2 (abcam, ab192022, clone EPR16266; 1:200 dilution); anti-Oma1 (SCBT, sc-515788, clone H-11; 1:50 dilution), anti-ATF4 (CST, 11815S, clone D4B8; 1:50

dilution), and anti-Cofilin (CST, 5175T, clone D3F9; 1:10,000 or 1:30,000 dilution).

Antibodies used for immunohistochemistry: anti-CD45 (abcam, ab10558, 1:1500 dilution), anti-Ki67 (abcam, ab16667, clone SP6; 1:100 dilution), anti-proSftpC (Millipore, AB3786; 1:500 dilution), anti-Podoplanin (abcam, ab11936, clone RTD4E10; 1:2000 dilution).

Antibodies used for cell isolation: anti-mouse biotin-conjugated CD45 (BD Biosciences, 553078, clone 30-F11), anti-mouse biotin-conjugated CD31 (BD Biosciences 553371, clone MEC 13.3), anti-mouse biotin-conjugated CD16/CD32 (BD Biosciences 553143, clone 2.4G2), and anti-mouse EpCAM microbeads (Miltenyi Biotec, 130-105-958) without dilution.

Validation

The antibodies used in this study were tested by the manufacturer.
-anti-Vinculin (abcam, ab129002, clone EPR8185). This antibody can be found in 206 citations. The manufacturer also provides antibody testing data: https://www.abcam.com/vinculin-antibody-epr8185-ab129002.html

-anti-NDUFS2 (abcam, ab192022, clone EPR16266). This antibody can be found in 3 citations. The manufacturer also provides antibody testing data and knockout validation: https://www.abcam.com/ndufs2-antibody-epr16266-ab192022.html

-anti-Oma1 (SCBT, sc-515788, clone H-11). This antibody can be found in 31 citations. The manufacturer also provides antibody testing data: https://www.scbt.com/p/oma1-antibody-h-11

-anti-ATF4 (CST, 11815S, clone D4B8). This antibody can be found in 661 citations. The manufacturer also provides antibody testing data: https://www.cellsignal.com/products/primary-antibodies/atf-4-d4b8-rabbit-mab/11815

-anti-Cofilin (CST, 5175T, clone D3F9). This antibody can be found in 245 citations. The manufacturer also provides antibody testing data: https://www.cellsignal.com/products/primary-antibodies/cofilin-d3f9-xp-rabbit-mab/5175

-anti-CD45 (abcam, ab10558). This antibody can be found in 282 citations. The manufacturer also provides antibody testing data: https://www.abcam.com/cd45-antibody-ab10558.html

-anti-Ki67 (abcam, ab16667, clone SP6). This antibody can be found in 1744 citations. The manufacturer also provides antibody testing data and knockout validation: https://www.abcam.com/ki67-antibody-sp6-ab16667.html

-anti-proSftpC (Millipore, AB3786). This antibody can be found in 16 citations. The manufacturer also provides antibody testing data: https://www.emdmillipore.com/US/en/product/Anti-Prosurfactant-Protein-C-proSP-C-Antibody,MM_NF-AB3786

-anti-Podoplanin (abcam, ab11936, clone RTD4E10). This antibody can be found in 65 citations. The manufacturer also provides antibody testing data: https://www.abcam.com/podoplanin--gp36-antibody-rtd4e10-bsa-and-azide-free-ab11936.html

-anti-mouse biotin-conjugated CD45 (BD Biosciences, 553078, clone 30-F11). This antibody can be found in 4 citations. The manufacturer also provides antibody testing data: https://www.bdbiosciences.com/en-us/products/reagents/flow-cytometry-reagents/research-reagents/single-color-antibodies-ruo/biotin-rat-anti-mouse-cd45.553078

-anti-mouse biotin-conjugated CD31 (BD Biosciences 553371, clone MEC 13.3). This antibody can be found in 14 publications. The manufacturer also provides antibody testing data: https://www.bdbiosciences.com/en-us/products/reagents/flow-cytometry-reagents/research-reagents/single-color-antibodies-ruo/biotin-rat-anti-mouse-cd31.553371

-anti-mouse biotin-conjugated CD16/CD32 (BD Biosciences 553143, clone 2.4G2). This antibody can be found in 15 citations. The manufacturer also provides antibody testing data: https://www.bdbiosciences.com/en-lu/products/reagents/flow-cytometry-reagents/research-reagents/single-color-antibodies-ruo/biotin-rat-anti-mouse-cd16-cd32.553143

-anti-mouse EpCAM microbeads (Miltenyi Biotec, 130-105-958). The antibody can be found in 5 publications. The manufacturer provides antibody testing data: https://www.miltenyibiotec.com/US-en/products/cd326-epcam-microbeads-mouse.html#130-105-958

Eukaryotic cell lines

Policy information about cell lines

Cell line source(s)

MLE-12 and 293T were purchased from ATCC.

Authentication

Neither of the cell lines used were authenticated.

Mycoplasma contamination

Cell lines tested negative for mycoplasma contamination. Cells were checked periodically.

Commonly misidentified lines
(See ICLAC register)

These cell lines are not listed in the database of commonly misidentified cell lines maintained by ICLAC.

Animals and other organisms

Policy information about studies involving animals; ARRIVE guidelines recommended for reporting animal research

Laboratory animals

Genetically modified mice on C57Bl/6J background were used. The age of the mice used in this study is ranged from newborn to 25-month-old, and specified in figure legends for each experiment. Both male and female mice were used in all experiments. Animals were housed at Northwestern University animal facility, where the animals were on a 14-h on, 10-h off light cycle, room temperature

range was 21-23°C, and humidity was within 30-70 % range compliant to the guidelines.
Ndufs2 floxed mice were genotyped using the following primers: Forward 5' - ATAAGAGTGGATAGGATGTTT - 3' ; flox reverse 5' - CATTTCTCCCTTCCCGTC - 3' ; and null reverse 5‘-AGTGGCAGAACAATAGAGTGATCCAGGG-3‘
Sdhd floxed mice were genotyped using the following primers: Sdhd Forward 5’ - GGAAGGCTCCAAGGGTGCAG - 3’ ; and Sdhd Reverse 5’ - CACATACACGCAGGCACTGG - 3’
SFTPC-Cre mice were genotyped using the following primers: Cre Forward: 5’-GCAGAACCTGAAGATGTTCGCGAT-3’ ; Cre Reverse: 5’-AGGTATCTCTGACCAGAGTCATCC-3’ ; Internal Control Forward: 5’-CTAGGCCACAGAATTGAAAGATCT-3’ ; and Internal Control Reverse: 5’-GTAGGTGGAAATTCTAGCATCATCC-3’
NDI1-LSL mice were genotyped using the following primers: Rosa26 Fwd 5’ – GAGTTCTCTGCTGCCTCCTG; Rosa26 Rev 5’ – CCGACAAAACCGAAAATCTG; and WPRE B Fwd 5’ – GACGAGTCGGATCTCCCTTT.
ROSA26Sor CAG-tdTomato mice were genotyped using the following primers: 5’-GGC ATT AAA GCA GCG TAT CC-3’ ; 5’-CTG TTC CTG TAC GGC ATG G-3’ ; 5’-CCG AAA ATC TGT GGG AAG TC-3’ ; and 5’-AAG GGA GCT GCA GTG GAG TA-3’

Wild animals	This study did not involve wild animals.
Field-collected samples	This study did not involve samples collected from the field.
Ethics oversight	All mouse work was done in accordance with Northwestern University Institutional Animal Care and Use Committee (IACUC).

Note that full information on the approval of the study protocol must also be provided in the manuscript.
